# No fixed item limit in visuospatial working memory

**DOI:** 10.1016/j.cortex.2016.07.021

**Published:** 2016-10

**Authors:** Sebastian Schneegans, Paul M. Bays

**Affiliations:** University of Cambridge, Department of Psychology, Cambridge, UK

**Keywords:** Working memory, Resource, Spatial, Variability, Bayesian

## Abstract

Investigations of working memory capacity in the visual domain have converged on the concept of a limited supply of a representational medium, flexibly distributed between objects. Current debate centers on whether this medium is continuous, or quantized into 2 or 3 memory “slots”. The latter model makes the strong prediction that, if an item in memory is probed, behavioral parameters will plateau when the number of items is the same or more than the number of slots. Here we examine short-term memory for object location using a two-dimensional pointing task. We show that recall variability for items in memory increases monotonically from 1 to 8 items. Using a novel method to isolate only those trials on which a participant correctly identifies the target, we show that response latency also increases monotonically from 1 to 8 items. We argue that both these findings are incompatible with a quantized model.

## 1 Introduction

Variability of error in recall from visual working memory increases monotonically with the number of items presented (Bays and Husain, 2008, Palmer, 1990, Wilken and Ma, 2004). Items with high physical or behavioral salience are recalled with enhanced precision, but at a cost to other items held simultaneously in memory, which are remembered with reduced fidelity (Bays et al., 2011, Gorgoraptis et al., 2011, Lara and Wallis, 2012, Melcher and Piazza, 2011). These results have led to the characterization of working memory as a limited resource that can be flexibly distributed between items: the more resource an item receives, the greater the resolution of its storage (Bays et al., 2009, Bays and Husain, 2008, van den Berg et al., 2012, Fougnie et al., 2012, Ma et al., 2014).

Zhang and Luck (2008), extending the model of Luck and Vogel (1997), proposed that the medium of working memory is instead quantized into a small number of “slots”. Each slot can hold the features of a single object in a bound representation. According to their analysis, human observers typically possess two or three slots. These slots are distributed among items in a resource-like way, such that multiple slots can be dedicated to the same object. When the number of slots exceeds the set size, e.g., when only one item is presented, the brain creates multiple independent representations of the same object. These independent representations are then averaged together at recall to generate a single estimate of the item's features. This aspect of the model is necessary to account for the experimentally-observed changes in representational precision with set size. Because slots can be allocated unevenly between objects, this model even permits a limited amount of flexibility in how precisely different items are stored, similar to a resource model.

To support their *slots* + *averaging* model, Zhang & Luck fit response data from a color recall task with a mixture of two components: a (circular) normally-distributed component of error centered on the true value of a probed item, and a uniformly-distributed component intended to capture guesses. The model predicts that, when the number of items is equal to or greater than the number of slots, every item either has one slot or no slots. As a result, responses should either be random, or distributed about the target with a single fixed variability. Consistent with this, Zhang & Luck observed that the width of the fitted normally-distributed component reached a plateau at around 3 items. The frequency of uniformly-distributed responses corresponded to at most 2–3 items stored.

Zhang & Luck considered only one source of error in their analysis, i.e., variability in memory for color. However, the color recall task required participants to use memory for location as well, to identify which one of the items in memory to report. Assuming location memory is also subject to variability, observers are expected to make “swap” errors on some trials, in which they incorrectly report one of the other items in the memory array. Critically, these errors look like random guesses under Zhang & Luck's analysis. Bays et al. (2009) showed that swap errors were indeed prevalent, and once taken into account, the frequency of random responses was substantially reduced and no longer consistent with a fixed item limit of any size.

The neural basis of the behaviorally observed capacity limits in visual working memory has been investigated using functional magnetic resonance imaging (fMRI) and electroencephalography (EEG) studies (Anderson et al., 2011, Todd and Marois, 2004, Xu and Chun, 2006). Several regions in prefrontal and posterior parietal cortex have been shown to exhibit a specific, sustained increase in blood oxygen level dependent (BOLD) signal associated with maintenance of items in working memory. Moreover, the strength of this signal has been found to increase with memory load, and the BOLD signal strength has been described as reaching a plateau at a set size of around four items (Todd & Marois, 2004). This has previously been interpreted as support for slot models, suggesting that the strength of the BOLD signal reflects the number of filled slots. This view is not compatible, however, with the *slots* + *averaging* model, which assumes that all slots are used even at lower set sizes to hold multiple copies of memory items.

More recent fMRI studies have used multivariate pattern analysis to decode the features held in working memory from the BOLD signal (Emrich et al., 2013, Riggall and Postle, 2012). The results showed that features could be decoded successfully from posterior parietal areas of cortex that did not show an overall increase in BOLD signal, but not from regions that featured elevated delay activity. This suggests that an elevated delay activity is not directly associated with coding of object features, and may instead reflect other aspects of the working memory task such as control of attention. Moreover, Emrich et al. (2013) and Sprague, Ester, and Serences (2014) showed that the precision with which memorized features could be decoded from fMRI data decreased significantly with increasing memory load. This finding is consistent with the idea of a limited resource being distributed among memory items. This possible link between neural activity and observed capacity limits in working memory tasks has been made explicit by a recent model which proposed that the limited resource is the number of action potentials generated in a neural population (Bays, 2014). The model has shown that decoding from a population representation with random noise can account for the specific distribution of response errors in working memory tasks, and that the effect of set size on memory performance can be explained by assuming that the total spiking activity of the population is normalized.

The present study introduces response latency as a new source of evidence from reproduction tasks to distinguish slot and resource models. Resource models make a link between the amount of resource allocated to an item and the rate of accumulation of evidence that determines when a response is generated: this predicts that response latency will increase monotonically with set size (Bays, 2015, Pearson et al., 2014). In contrast, the slot model holds that the memory state can be fully characterized by the allocation of the available slots to different items (Zhang & Luck, 2008). This implies that items that are out of memory, i.e., did not get a slot, cannot influence recall of items in memory. Therefore, if analysis is restricted to those items that received a slot (according to the model) performance parameters such as latency must reach a plateau at the point when every item has either one or zero slots, i.e., when the number of items equals the number of slots. This is because further increases in set size will only change the number of items with zero slots, leaving the status in memory of items with a slot unchanged.

Here we directly examined the precision and latency of working memory for location, using a pointing task in two-dimensions. Consistent with previous results, we found that swap errors were prevalent and guessing rare. Inconsistent with the *slots* + *averaging* model, variability for items in memory increased monotonically from 1 to 8 items, with no sign of a plateau. We introduce a novel analytical method to explicitly identify trials on which participant's responses were drawn from the error component centered on the target. Examining response latency for these trials alone we observed a monotonic increase in latency from 1 to 8 items, again inconsistent with the *slots* + *averaging* model.

## 2 Experimental procedures

### 2.1 Experiment

**Fig. 1:**
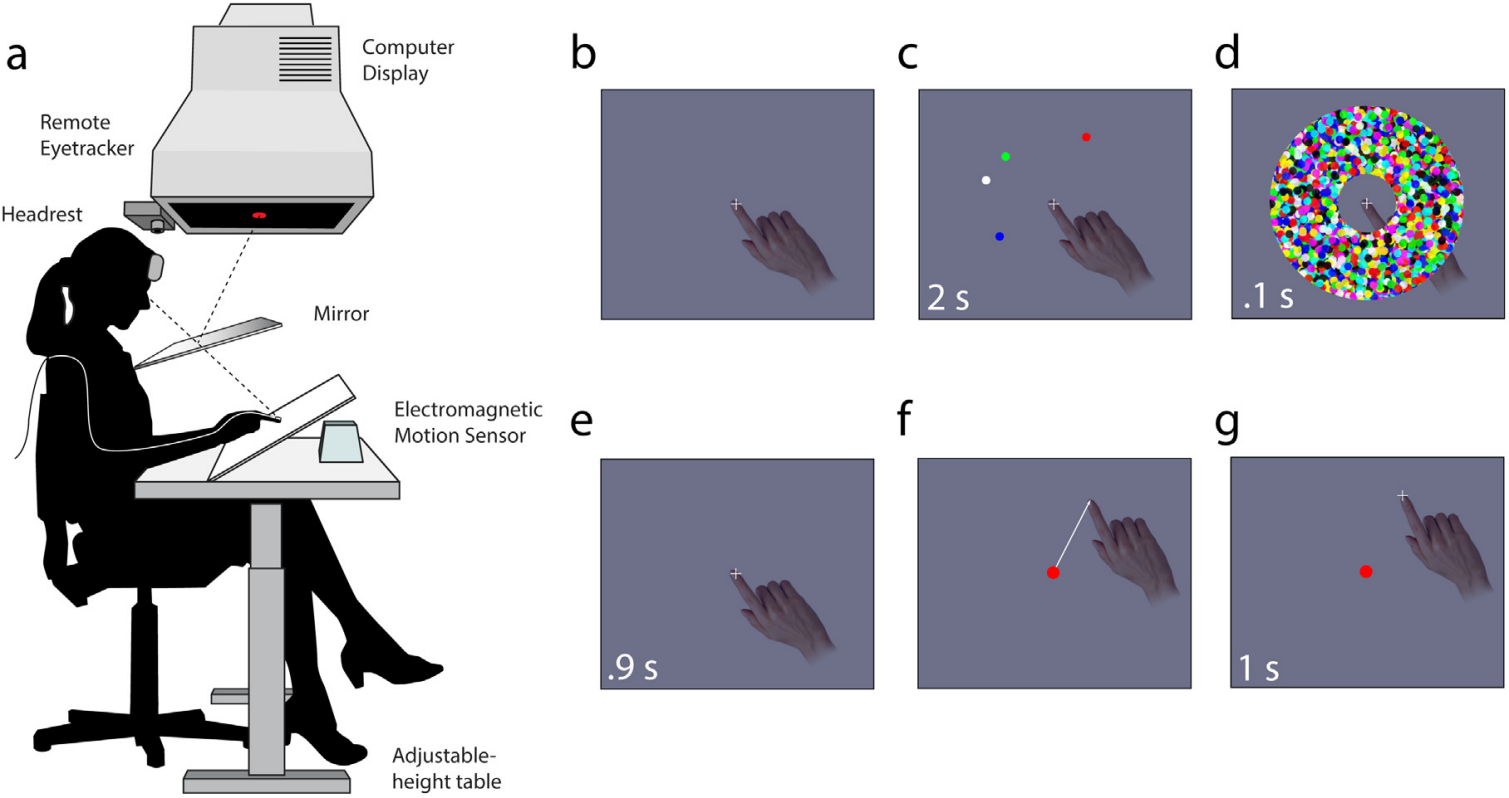
Spatial working memory task. (a) Participants' hand and gaze positions were recorded while they viewed a stimulus display reflected into the plane of the hand. (b–g) On each trial, a sample array was presented consisting of one to eight discs with randomly-chosen colors and locations, followed by a mask. After a delay, a single color was presented and the participant moved their fingertip to the corresponding remembered position.

Eight participants (five males, three females; aged 21–26 years) participated in the study after giving informed consent, in accordance with the Declaration of Helsinki. All participants were right-handed, reported normal color vision and had normal or corrected-to-normal visual acuity. Participants sat with their head supported by a forehead and chin rest and their right index finger touching an inclined surface (see Fig. 1a). Index fingertip position was monitored online at 133 Hz using an electromagnetic motion sensor (Ascension Technology Corporation). Stimuli were presented on a 21-inch CRT monitor with a refresh rate of 130 Hz. Participants viewed the reflection of the monitor in a semi-silvered mirror, angled such that the stimuli appeared to lie in the plane of the inclined surface. Eye position was monitored online at 1000 Hz using an infrared eye tracker (SR Research).

Each trial began with the presentation of a central black cross (diameter .75° of visual angle) against a gray background. Subjects were required to fixate the cross and position their fingertip at the cross's location. Once stable gaze and fingertip positions were recorded within 2° of the cross, a sample array consisting of between one and eight colored discs (.5° radius) was presented for 2 sec (see Fig. 1c for an illustration). Each disc's location was selected uniformly at random from the annular space 5° to 10° from the cross. Every disc was separated from its neighbors by a minimum of 1.5°. Colors were chosen at random from a set of eight highly-discriminable colors.

The sample array was followed by a brief pattern mask (100 msec, see Fig. 1d) and then the display was blanked for 900 msec. A probe display was then shown consisting of a single central disc (.5° radius), matched in color to one of the discs (the *target*) chosen at random from the sample array. Participants were instructed to make a single movement of their hand to bring the fingertip to the location they remembered corresponding to the probe color, keeping contact with the surface throughout the movement. Subjects were instructed to be as precise as possible; speed was not emphasized. Once the participant had completed the movement, a cross was displayed at the position of the fingertip for 1 sec. Each subject completed 320 trials in total, comprising 80 trials with each of the set sizes {1, 2, 4, 8}.

### 2.2 Analysis

Response latency for each trial was calculated as the time from probe presentation until fingertip velocity first exceeded 5°/sec. Trials with latency less than 200 msec were excluded from further analysis (3.7% of trials). The response position was calculated as the fingertip location at the end of the movement when velocity fell below 3°/sec for >250 msec. The response error was defined as the Euclidean distance between the response position and the target location.

To test for an influence of non-target items on responses, we computed an expected distribution of response deviations from non-targets under the assumption that non-target items did not affect responses. For each trial, we generated 1000 new random spatial configurations of non-targets, following the same constraints for stimulus locations as in the experiment, and with locations selected to match the distribution of target to non-target distances within each set size. We then calculated deviations of responses from these simulated non-targets and compared their distribution to the actual distribution of deviations obtained in the experiment.

**Fig. 2:**
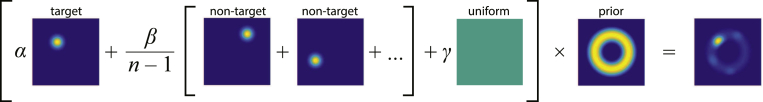
Components of the mixture model for generating response positions. The tiles show color-coded probability distributions for the response location over two-dimensional space. The mixture model consists of a normal distribution centered on the target location, normal distributions centered on each non-target item location to account for swap errors, and a uniform distribution to account for random guessing. The weighted sum of these components is multiplied with a distribution that reflects the expectation of possible target positions given that item locations in the task were restricted to a fixed annulus around the fixation point. The resulting probability distribution for the response position in an example trial is shown in the rightmost tile.

**Fig. 3:**
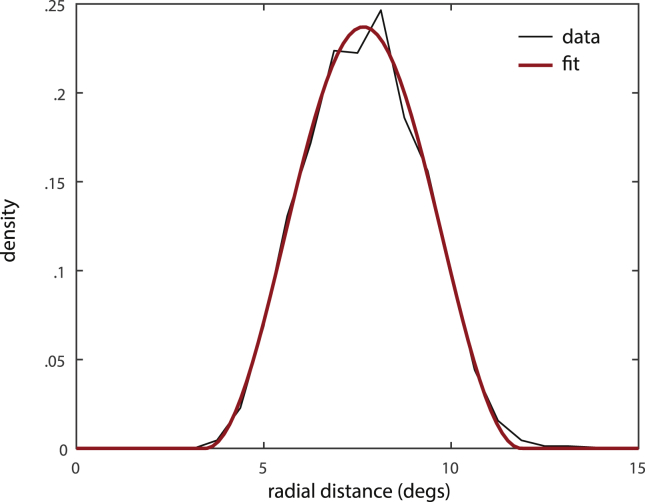
Response position eccentricity and approximating function. The distribution of response eccentricities (radial distance from the fixation point) over all subjects is shown in black; the function $h\left(\hat{x}\right)$ used to approximate this distribution is shown in red. The function $h\left(\hat{x}\right)$ was used to capture participant's prior beliefs about likely stimulus locations in the mixture model.

We fit a probabilistic model to the data that attributed the distribution of responses to a mixture of three components (illustrated in Fig. 2) corresponding to: reporting the target location, mistakenly reporting one of the other (non-target) locations in the memory array, and responding at random. The model is described by the following equation:

(1) $$p\left(\hat{x}\right)=\alpha f\left(\hat{x},x\right)+\beta \frac{1}{M}\sum_{i}^{M}f\left(\hat{x},y_{i}\right)+\gamma h\left(\hat{x}\right)$$

where $\hat{x}$ is the response location, ***x*** is the target location, and $\left\{y_{1}\ldots y_{M}\right\}$ are the *M* non-target locations. The probability of correctly reporting the target item is given by *α*; the probability of incorrectly reporting a non-target item by *β*; and the probability of responding at random by *γ* = 1 − *α* − *β*. The distribution of responses around target and non-target items was modeled as a bivariate normal with standard deviation *σ*, multiplied by a distribution $h\left(\hat{x}\right)$ intended to reflect participants' beliefs as to where items were constrained to appear in the display:

(2) $$f\left(\hat{x},x\right)=\frac{1}{Z}\phi^{2}\left(\frac{\hat{x}-x}{\sigma }\right)h\left(\hat{x}\right)$$

where $\phi^{2}$ is the unit spherical bivariate normal density function, i.e., $\phi^{2}\left(x\right)=\frac{1}{2\pi }e^{-\left\|x^{2}\right\|/2}$, and *Z* is a normalization term chosen such that $\iint_{{\mathbb{R}}^{2}}f\left(\hat{x},x\right)d\hat{x}=1$. For distribution $h\left(\hat{x}\right)$, a function was chosen that approximated the observed distribution of response eccentricities, pooled across all subjects and trials (see Fig. 3):

(3) $$h\left(\hat{x}\right)=\{\begin{array}{ccc} \frac{1}{2\pi w\left|\left|\hat{x}\right|\right|}\left(\text{cos}\left(\frac{2\pi }{w}\left(\left|\left|\hat{x}\right|\right|-\mu \right)\right)+1\right) & \text{if} & |\left\|\hat{x}\right\|-\mu |\leq w/2\\ 0 & \text{if} & |\left|\left|\hat{x}\right|\right|-\mu |>w/2 \end{array}$$

A very few responses (.5% of trials) had zero probability under the distribution $h\left(\hat{x}\right)$; these trials were excluded from analysis.

Maximum likelihood (ML) estimates of the parameters *α*, *β*, *γ*, *σ* were obtained separately for each subject and set size using a nonlinear optimization algorithm (*fminsearch* in MATLAB). As well as the full model described by Eq. (1), we also fit reduced models in which one or both of *β* and *γ* were set to zero. Models were compared using the Akaike Information Criterion, with a correction for finite sample size (AICc). Predictions of error distributions under the best-fitting model were generated using Monte Carlo simulation (>10^6^ samples).

We calculated the probability *P*(*T*) that a response on a given trial was drawn from the target-centered mixture component, based on ML parameters $\hat{\theta }$ obtained from the best-fitting model, using Bayes theorem:

(4) $$P\left(T|\hat{x},\hat{\theta }\right)=\frac{p\left(\hat{x}|T,\hat{\theta }\right)P\left(T|\hat{\theta }\right)}{p\left(\hat{x}|\hat{\theta }\right)}=\frac{\hat{\alpha }f\left(\hat{x},x\right)}{\hat{\alpha }f\left(\hat{x},x\right)+\hat{\beta }\frac{1}{M}\sum_{i}^{M}f\left(\hat{x},y_{i}\right)+\hat{\gamma }h\left(\hat{x}\right)}$$

We fit response times, *t*, with the following equation:

(5) $$p\left(t|N\right)=\phi^{-1}\left(t-t_{0};\mu_{N},\lambda_{N}\right)$$

where $\phi^{-1}$ is the inverse Gaussian probability density function and *N* is the set size. This is equivalent to a drift diffusion model (Ratcliff & Rouder, 1998) with unit threshold, non-decision time *t*_0_, drift 1/*μ*_*N*_ and variance 1/*λ*_*N*_. Data from each subject were fit separately with parameters {*t*_0_, *μ*_1_, *μ*_2_, *μ*_4_, *μ*_8_, *λ*_1_, *λ*_2_, *λ*_4_, *λ*_8_}.

We also examined a non-normal model of response generation in which the distribution of responses around target and non-target items was modeled as the product of stable distributions with zero mean (*δ* = 0) and skewness (*β* = 0), leaving scale (*γ*) and shape (*α*) as free parameters.

### 2.3 Replication study

We performed a replication study to test whether the results generalized to other probe feature dimensions, specifically shape. Eight participants (one male, seven females; aged 18–28 years) participated in the experiment after giving informed consent. All participants were right-handed and had normal or corrected-to-normal visual acuity. Experimental setup and procedure were identical to the original experiment, except for the stimuli used in the sample array and as probe: Stimuli were chosen from eight different white shapes (Supplementary Fig. 1), scaled to have an equal surface area of .75° of visual angle squared (diameters 1.0°–1.5°). The pattern mask was adjusted to match the stimulus features. Each subject completed 320 trials in total, comprising 80 trials with each of the set sizes {1, 2, 4, 8}.

## 3 Results

We investigated the effect of memory load on visual working memory precision and response latencies in a manual response task. Participants viewed an array of differently-colored discs; after a blank retention interval, they were cued with the color of one of the discs (the target), and required to make a pointing movement with their index finger to its memorized location.

**Fig. 4:**
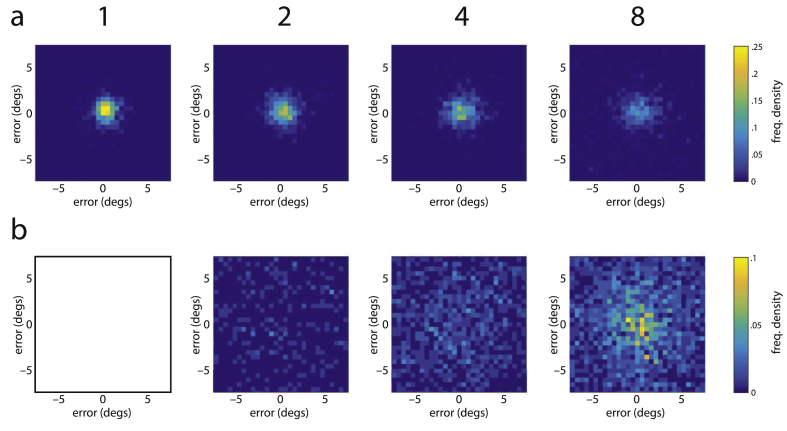
Distribution of response positions relative to target and non-target items for different set sizes. (a) Frequency density of response positions relative to target locations over two-dimensional space. (b) Frequency density of response positions relative to the locations of non-target items.

**Fig. 5:**
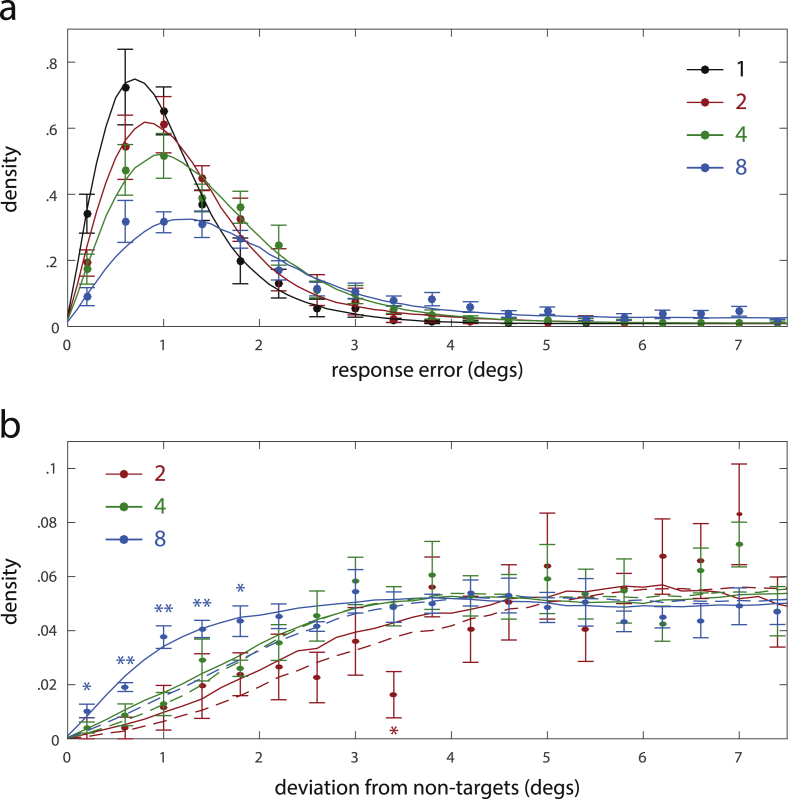
Distribution of response errors and deviations from the locations of non-target items for different set sizes. (a) Frequency density of response errors (Euclidean distance from target location). (b) Frequency densities of deviations from non-target locations. Dots show mean frequency density for different error magnitudes, error bars show standard error over subjects. Dashed lines show the expected frequency densities under the assumption that non-target locations do not influence the response position; asterisks indicate significant deviations of empirical frequency from expected values (*p < .05; **p < .01). Simulation results for the best-fitting mixture model are plotted as solid lines.

Fig. 4a shows the distribution of response positions relative to the target location for different set sizes (number *N* of items in the memory display). The distribution is narrowly concentrated on the actual target location for set size *N* = 1, but becomes more dispersed with increasing set size. A repeated measures one-way analysis of variance (ANOVA) showed a significant effect of set size on mean response error [*F*(3, 28) = 47.78, *p* < .001]. The increase of response error with set size was well captured by a linear model (R^2^ = .954). Subsequent *t*-tests showed a significant increase in mean response error with every increase in set size [from *N* = 1 to *N* = 2: t(7) = 2.47, *p* = .043; from *N* = 2 to *N* = 4: t(7) = 4.34, *p* = .003; from *N* = 4 to *N* = 8: t(7) = 17.61, *p* < .001]. The distribution of response errors (Euclidean distance from target) for each set size is shown in Fig. 5. The distributions become wider as set size increases, with a higher proportion of large response errors at larger set sizes.

### 3.1 Evidence for swap errors

Previous work suggests that swap errors make a significant contribution to responses in working memory reproduction tasks (Bays et al., 2009). In the context of the present study, a swap error occurs when a subject reports the location of a memory item other than the target. To test whether swap errors occurred, we analyzed the distribution of response positions relative to the locations of non-target items in the memory display. This distribution is shown for different set sizes in Fig. 4b. For a set size of *N* = 8, we see a distinct concentration of response positions around the locations of non-target items.

To confirm that this response pattern is indeed evidence for swap errors, we analyzed the distribution of response deviations from non-targets (Euclidean distance between the response position and the locations of each non-target item), shown in Fig. 5b. We compared this distribution to a baseline distribution of deviations that would be expected if non-target locations had no influence on the response, shown as dashed lines. For set size *N* = 8, the observed distribution of response deviations from non-targets differs significantly from this baseline distribution, with a higher proportion of trials showing deviations less than 2°. This confirms that responses are indeed concentrated around the locations of non-targets in a fashion that cannot be explained by random guessing, and provides strong evidence for the occurrence of swap errors.

### 3.2 Mixture model

In order to estimate the contributions of swap errors and random guesses to the observed pattern of response positions, we fit the response position data with a mixture model (Bays et al., 2009). The model contains three components, shown in Fig. 2: a normal distribution around the target location; normal distributions around all non-target locations to reflect swap errors; and a uniform distribution to reflect random guessing. The result of this mixture model is additionally scaled with a distribution that reflects participants' expectations about possible target locations. This scaling is employed since the actual target location is restricted to an annulus within the two-dimensional response space: we judged that participants were unlikely to respond substantially outside this annulus even if they were making a random guess. The distribution of observed response eccentricities shown in Fig. 3 indeed suggests that participants incorporated an expectation of possible target locations into their response generation, and the profile of the expectation distribution in the model is fit to this observed distribution (see Methods for full details).

**Fig. 6:**
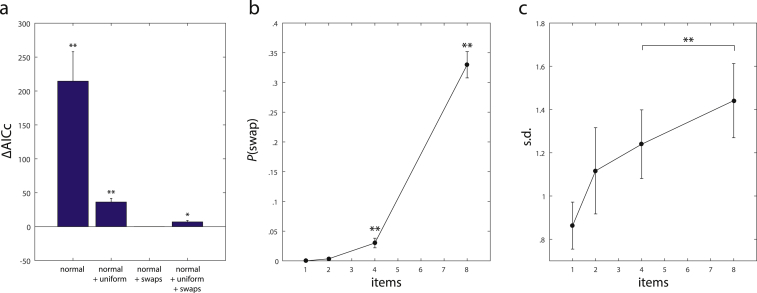
Model comparison and parameters of mixture model fit. (a) AICc scores of different mixture model variants relative to the score of the best-fitting model. (b) Estimated proportions of swap errors in best-fitting mixture model for different set sizes. (c) Estimated standard deviations of normal distribution in best-fitting mixture model.

In addition to the three component mixture model, we also generated reduced models in which the swap component, the uniform component, or both of them were omitted. We compared the different mixture models using their AICc scores, shown in Fig. 6a.

The normal distribution around the target location alone provides a relatively poor fit of the data: including swap errors and/or random guessing in the mixture model leads to lower AICc scores, indicating a better fit of the data even when adjusted for the higher number of free parameters. The lowest AICc score was achieved by a model that allows for swaps, but not random guessing, and the score of this model over all subjects and set sizes was significantly lower than the scores of other model variants [normal distribution with swap errors compared to normal distribution only: t(7) = 4.94, *p* = .002; compared to normal distribution with random guessing: t(7) = 6.65, *p* < .001; compared to normal distribution with swap errors and random guessing: t(7) = 3.26, *p* = .014; *t*-tests performed after finding no significant deviations from normality in AICc scores for each model using Lilliefors test]. Moreover, even in the model variant with both swap errors and random guesses, the estimated proportion of guesses was very low (<.061 for all subjects and set sizes), and not significantly different from zero for any set size [*N* = 2: t(7) = 1.55, *p* = .16; *N* = 4: t(7) = 1.00, *p* = .35; *N* = 8: t(7) = 2.10, *p* = .074].

These results indicate that human performance in this task can be best explained as a combination of reporting the target location and erroneously reporting the location of a non-target. We used the corresponding mixture model to simulate the distribution of response errors and response deviations from non-targets in the experiment. The model provides a good quantitative fit of the empirical results for all set sizes (solid lines in Fig. 5a and b).

The selected mixture model has two free parameters: the proportion of swap errors, and the standard deviation of the normal distributions. Fig. 6b and c shows how these parameters change with set size. We found a significant increase in the estimated proportion of swap errors from set sizes *N* = 2 to *N* = 8 in a repeated measures ANOVA [*F*(2, 21) = 176.69, *p* < .001; set size *N* = 1 is excluded from this analysis since no swap errors can occur]. For set size *N* = 2, the estimated proportion of swap errors was not significantly different from zero [t(7) = 1.5261, *p* = .171], but significant proportions of swap errors occurred at set size *N* = 4 [t(7) = 3.69, *p* = .008] and *N* = 8 [t(7) = 14.96, *p* < .001]. However, this increase in swap errors was not the only factor that accounted for the empirically observed increase in mean response error. The standard deviation of the normal distribution likewise increased monotonically with set size, and the increase is partially captured by a linear model (R^2^ = .881). Specifically, we found a significant increase of this parameter between set sizes *N* = 4 and *N* = 8 [t(7) = 4.69, *p* = .002]. This is of particular interest since it directly contradicts a prediction of the *slots* + *averaging* model proposed by Zhang and Luck (2008): based on this model, the standard deviation should plateau for set sizes greater than 2 or 3.

### 3.3 Response latencies

**Fig. 7:**
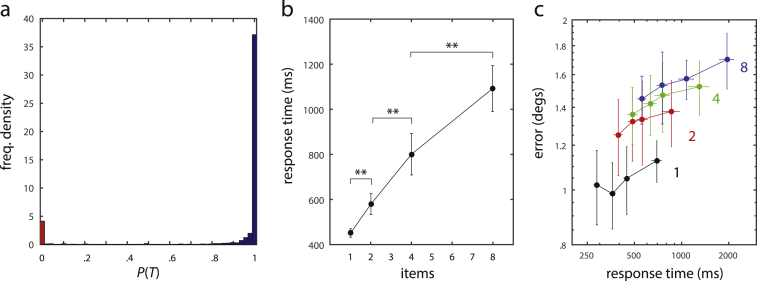
Analysis of response latencies. (a) Distribution of probabilities *P*(*T*) that the response of a trial was directed towards the target disc. Trials with *P*(*T*) > .5 are classified as responses to target (blue), all other trials as swap errors (red). (b) Mean response times for different set sizes. Error bars indicate the standard error over subjects. (c) Mean response errors plotted against response times. Trials are quantilized by response time. Filled circles show mean response latency and mean response error for each quantile, error bars indicate standard error in both dimensions.

For the analysis of response latencies, we focus on those trials in which the response was made towards the target location and exclude trials in which swap errors occurred. In this way, we avoid any confounding effects that the different proportions of swap errors may have, given that the distribution of response latencies may be different on swap trials. We determined for each individual trial the probability *P*(*T*) that the response was directed towards the target location (rather than the location of a non-target), using Bayes' theorem in combination with the parameters of the best-fitting mixture model. Fig. 7 shows the histogram of obtained probabilities *P*(*T*). The distribution is clearly bimodal with probability values clustered around 0 and 1, indicating that this approach allows an unambiguous classification for a large majority of trials. We classified all trials with *P*(*T*) > .5 as responses towards the target, and excluded all other trials from the response time analysis (8.8% of trials).

**Fig. 8:**
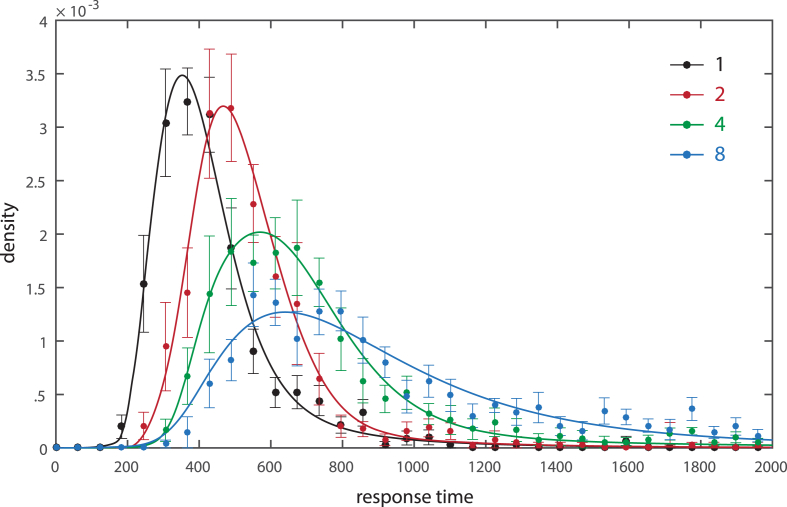
Distribution of response latencies for different set sizes. Filled circles show the mean frequency density of different response latencies, with error bars indicating the standard error across subjects. Fits of a drift diffusion model are shown as solid lines.

Fig. 8 plots distributions of latency for responses directed towards the target at each set size. Consistent with the description of working memory retrieval as a stochastic decision process (Pearson et al., 2014), we found that a drift diffusion model (colored curves) provided a close fit to the empirically observed distributions of response latency.

Mean response times for target-centered responses at each set size are shown in Fig. 7b. A repeated measures one-way ANOVA revealed a significant effect of set size on response latency [*F*(3, 28) = 14.70, *p* < .001]. The increase in latency is partially explained by a linear model (R^2^ = .984). Subsequent *t*-tests showed a significant increase in response time for each increase in set size [*N* = 2 *vs* *N* = 1: t(7) = 3.87, *p* = .006; *N* = 4 *vs* *N* = 2: t(7) = 4.52, *p* = .003; *N* = 8 *vs* *N* = 4: t(7) = 8.11, *p* < .001]. This finding provides further evidence against the slot model. In that model, each available slot should be filled with a single memory item for both set sizes *N* = 4 and *N* = 8. Since we analyzed only trials in which the response was ultimately directed towards the target, the target item must have been among the stored items. The state of the slot model at the time of response generation would thus be identical for the two set sizes, and the presence or absence of additional items in the memory display could have no effect on response latencies.

Given that we found an increase in both response error and response latency with set size, the question arises whether these observations are independent effects of set size, or whether there is a more general correlation between precision and latency that is not driven by set size effects alone. To test this, we performed an ANCOVA on all trials classified as responses towards the target, with response error as dependent variable and response latency as continuous independent variable, controlling for set size. We did find a significant correlation between response error and latency that is not explained by set size effects [*F*(1, 3) = 21.86, *p* < .001]. An increase in magnitude of response errors with increasing latencies can be observed for all set sizes, as shown in Fig. 7c.

### 3.4 Non-normal error distributions

In the mixture models presented so far, we have assumed that the response positions around both target and non-target locations follow a normal distribution in two-dimensional response space, consistent with earlier mixture models of working memory (Bays et al., 2009, Zhang and Luck, 2008). However, recent research has shown that response errors in visual working memory tasks typically display systematic deviations from the normal distribution that cannot be explained by swap errors or random guessing (Bays, 2014, van den Berg et al., 2012, Fougnie et al., 2012). To test whether such deviations are present in the current study, we fit the response position data with a variant of the best-fitting mixture model, in which the normal distributions were replaced by stable distributions. The stable distribution has a shape parameter *α* as additional free parameter: it is identical to the normal distribution for *α* = 2 and varies progressively from the normal for *α* < 2, becoming more peaked with longer tails.

**Fig. 9:**
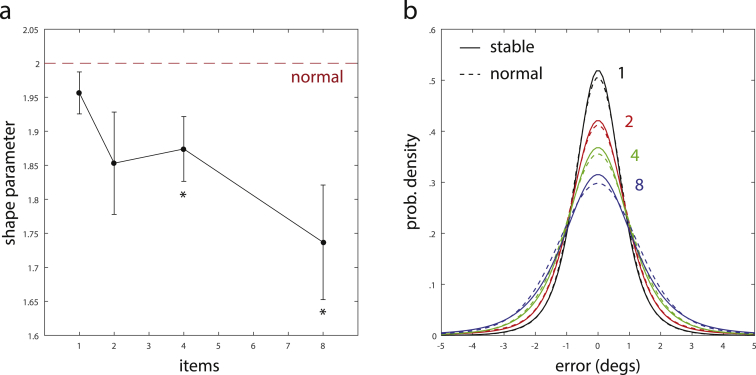
Mixture model fits with stable distributions. (a) Shape parameters of stable distributions in best-fitting mixture models. Filled circles show mean values for different set sizes, error bars indicate standard error over subjects. Dashed line indicates the shape parameter corresponding to normal distribution. (b) Comparison of stable distributions (solid lines) and normal distributions (dashed lines) in best-fitting mixture models. Curves for each set size are based on mean parameter values of model fits over all subjects.

The estimated shape parameters for different set sizes in these model fits are shown in Fig. 9a. The shape parameters were significantly different from *α* = 2 for set sizes *N* = 4 [t(7) = 2.65, *p* = .033] and *N* = 8 [t(7) = 3.12, *p* = .017], demonstrating a significant deviation from the normal distribution. Fig. 9b shows a direct comparison of the best-fitting normal and stable distribution for each set size, highlighting a small, but consistent deviation towards longer tails in the stable distributions. It is noteworthy that this deviation from the normal distribution is not an effect of swap errors, since these are explicitly accounted for in the mixture model, nor random guesses, since these were not observed in the present task.

While these results provide support for deviations from normality in the error distributions, the obtained AICc scores for the model fit with a stable distribution were slightly higher than for the best-fitting model with normal distributions, although the difference failed to reach significance [t(7) = 2.26, *p* = .059]. This indicates that the model with stable distributions, while yielding slightly higher likelihood values, does not provide a better explanation for the empirical data when taking into account the additional free parameter.

### 3.5 Replication study

In the replication study, we used shape instead of color as the probe dimension (Supplementary Fig. 1). Response position and latency data were analyzed in the same way as in the original experiment, and all key findings reported there were reproduced. In particular, we found a significant effect of set size on mean response error [*F*(3, 28) = 81.72, *p* < .001], with a significant increase in response error with every increase in set size. We found evidence for swap errors in the distribution of response positions relative to the positions of non-target items in the sample array. A specific concentration of responses around the positions of non-targets was observed for set size *N* = 8, and, unlike in the previous experiment, also for *N* = 4.

We obtained ML fits for the different versions of the mixture model based on the normal distribution. As in the previous experiment, the lowest AICc scores (best fits after adjustment for number of free parameters) were achieved by a model that included swap errors, but not random guessing (Supplementary Fig. 2a). The AICc scores for this model across subjects were significantly lower than the scores of each other model variant [compared to normal distribution only: t(7) = 8.92, *p* < .001; compared to normal distribution with random guessing: t(7) = 8.56, *p* < .001; compared to normal distribution with swap errors and random guessing: t(7) = 5.01, *p* = .002].

The estimated proportions of swap errors in the best-fitting model were overall higher than in the original experiment (consistent with the finding that swap errors were evident even at set size *N* = 4 in this experiment), with estimated proportions significantly greater than zero at set size *N* = 4 [t(7) = 3.98, *p* = .005] and *N* = 8 [t(7) = 16.98, *p* < .001; Supplementary Fig. 2b]. The estimated standard deviation of the normal distribution increased monotonically with set size, and in particular showed a significant difference between set sizes *N* = 4 and *N* = 8 [Supplementary Fig. 2c; t(7) = 2.59, *p* = .036], reproducing this critical finding from the previous experiment.

We analyzed response latencies for those trials which were determined to be directed at the position of the target (16.3% of trials excluded; Supplementary Fig. 3a), and found a significant effect of set size on mean response latency [*F*(3, 28) = 10.62, *p* < .001]. Critically, we again found a significant increase in response latency with every increase in set size, with no evidence for a plateau at higher set sizes [Supplementary Fig. 3b; *N* = 2 *vs* *N* = 1: t(7) = 5.75, *p* < .001; *N* = 4 *vs* *N* = 2: t(7) = 3.41, *p* = .011; *N* = 8 *vs* *N* = 4: t(7) = 3.39, *p* = .012]. The finding of a significant correlation between response error and latencies when controlling for set size was likewise reproduced in this experiment [Supplementary Fig. 3c; *F*(1, 3) = 13.86, *p* = .002].

When fitting the data with a mixture model using stable distributions, we did find a trend towards lower values of the parameter *α* at higher set sizes. Unlike in the original experiment, the deviation from *α* = 2 (normal distribution) was not significant at any set size, although it approached significance at set sizes *N* = 4 [t(7) = 2.23, *p* = .061] and *N* = 8 [t(7) = 2.03, *p* = .082]. Consistent with these low deviations from normality, the mixture model with normal distributions reached overall significantly lower AICc scores than the model with stable distributions [t(7) = 2.67, *p* = .032].

## 4 Discussion

Examining recall of object locations from visuospatial working memory, we observed that recall variability increased steadily with increasing number of to-be-remembered items. Two explanations have previously been put forward for this pattern. The *resource* hypothesis (Bays et al., 2009, Bays and Husain, 2008, van den Berg et al., 2012, Fougnie et al., 2012, Ma et al., 2014) proposes that all items are stored, but variability in their internal representations increases as the number of items in memory increases, because each item gets a smaller share of a fixed quantity of representational medium. The *slots* + *averaging* hypothesis (Zhang and Luck, 2008, Zhang and Luck, 2011) proposes that working memory holds only 2 or 3 internal representations (“slots”), each storing the features of a single object with fixed variability; that these representations can be of the same item, in which case they are averaged together at recall; and that probing an item without a slot results in a random guess.

Although both models predict a monotonic increase in error with set size, the composition of these errors is different. The resource model predicts two kinds of errors in the present task: noisy responses distributed around the location of the target (due to variability in memory for location) and noisy responses distributed around the locations of other, non-target items (swap errors, due to variability in memory for color; see below). Both target-centered variability and swap error frequency should increase monotonically with set size, according to this model. The *slots* + *averaging* model, on the other hand, predicts a mixture of noisy responses centered on the target and random guesses. Target-centered variability should increase (due to averaging) until the number of items is equal to the number of slots, then plateau; random responses should be present only when the number of items exceeds the number of slots, then should increase with set size.

To distinguish between these hypotheses, we fit models to the data comprising mixtures of target-centered errors, swap errors, and random guesses. We found no evidence for random guessing in this task: the best-fitting model incorporated only target-centered responses and swap errors. The variability of target-centered responses increased monotonically with set size up to 8 items, with no evidence for a plateau at 2 or 3 items. The prevalence of swap errors also increased monotonically with set size. These results favor the resource hypothesis.

Swap errors are an inevitable consequence of variability in recall for the feature dimension of the probe stimulus, in the present study either color or shape. When for instance a color probe is presented, the subject has to select that item from working memory whose remembered color is closest to this probe; but since all items' colors are remembered with a random error, there is a certain probability that a non-target item will be selected as the closest one instead of the target, and this non-target's location will consequently be reported. This account of swap errors (Bays et al., 2009) predicts that non-targets most similar to the target in the probe dimension are most likely to be the object of a swap error; this has indeed been demonstrated (Bays, 2016; see also Emrich and Ferber, 2012, Rerko et al., 2014). A previous study (Rajsic & Wilson, 2014) examined recall of object tangential position on a circle, and observed minimal guessing errors, consistent with the present results. In that study it was found that representing non-targets along with the probe stimulus substantially decreased the frequency of swap errors and increased the frequency of target-centered responses; this result is consistent with the hypothesis that swap errors are due to incorrectly selecting a non-target item from working memory for response generation: once non-targets are ruled out as possibilities by their presence in the probe display, this kind of error is effectively abolished.

Several previous studies that reported results in favor of the slot model did not take swap errors into account. Zhang and Luck (2011) observed that reducing the required precision with which four colored squares had be memorized did not increase the estimated number *K* of memorized items, and concluded that memory resources could not be flexibly distributed to create lower-quality representations of a larger number of items (see also He, Zhang , Li, & Guo, 2015). However, their estimate of *K* was based on a mixture model that did not allow for swap errors, and may therefore underestimate the number items actually held in working memory. It is then in particular possible that subjects actually formed memory representations for all items even in the high-precision condition, so that no further increase in the estimate of *K* could be expected by lowering the precision requirements. The authors further proposed that the apparent lack of a fixed item limit in other studies may be explained by chunking strategies, in which some information about multiple objects can be memorized by representing e.g., the average of their locations in a single slot. It is not clear, however, how such chunking could account for performance in the current task, in which subject not only have to memorize the locations of multiple objects, but also their distinct colors or shapes in order to select the target. Moreover, the chunking approach does not appear to account for the effect of set size on response latency, which we introduced here as an additional measure to distinguish between slot and resource models.

Pearson et al. (2014) showed that, in a working memory task with a two-alternative forced choice design (Bays & Husain, 2008), response latency increased with set size. The distribution of latencies was consistent with an evidence-accumulation process (Carpenter and Williams, 1995, Ratcliff, 1978, Usher and McClelland, 2001), suggesting that the rate of accumulation depends on the amount of resource allocated to the target item. However, this study could not distinguish between target-centered responses, swap errors and guessing, all of which may have different latency profiles. Here, we used a Bayesian method to identify only those trials on which the response was drawn from the target-centered distribution, thereby excluding swap errors (and guesses had there been any) from the analysis. The results demonstrate a monotonic increase in retrieval latency with set size, in this case in a reproduction task, as opposed to a forced-choice task. The latency distributions were well-described by a drift diffusion model of evidence accumulation (Gold and Shadlen, 2007, Ratcliff, 1978, Smith and Ratcliff, 2004).

We further showed that reaction time was correlated with error, with longer latencies associated with greater magnitudes of error within each set size. This mirrors results in perceptual decision tasks, in which error trials typically have longer latencies (Drugowitsch et al., 2012, Laming, 1968), and may in future prove an important criterion for discriminating between different models of working memory retrieval. We note that in the current tasks, subjects were only instructed to respond as precisely as possible, with no instructions or limitations for response time. Limiting the response time may significantly alter response behavior. Further studies will be necessary to determine how this affects response precision and proportion of swap errors.

Unlike the resource model, the *slots* + *averaging* model predicts plateaus for target-centered responses in performance metrics such as response latency. This is because, according to the model, once the number of items is equal to the number of slots, each item in memory has exactly one slot. Further increases in set size affect the proportion of items in memory, but not the number of slots per-item. Therefore, considering specifically target-centered responses, even if response latency depends on the number of slots allocated to the target item, it will not vary with set size once the 2–3 slot limit is exceeded. We found no evidence for such a plateau in our data, again consistent with the resource hypothesis.

While our findings are evidence against a capacity limit of a small number of items, they cannot rule out a *slots* + *averaging* model with arbitrarily high numbers of slots: indeed such a model would be theoretically indistinguishable from a resource model. One possibility, therefore, is that a larger number of slots is available for locations than for features like color, so that, in spatial recall tasks, a plateau in response time and response precision would only be reached at higher set sizes than tested here. However, such a separation of slots for different feature dimensions would constitute a major deviation from existing slot models, which assume that each slot holds a bound object representation. It would require the specification of a binding mechanism between slots for different features, and the associated mixture model would need to be extended to account for cases in which only a partial memory of an object is retained. Given that the flexible resource model has already been successful in explaining behavioral results in a wide range of working memory tasks, we believe that it currently provides the best explanation for our findings.

A recent neural model of working memory recall (Bays, 2014) proposed that the limited resource is spiking activity of neurons encoding stimuli in memory. In this model, feature values of memorized items are assumed to be encoded in the activity of a population of neurons with different tuning curves. Neural activity varies from trial-to-trial with a Poisson distribution, which leads to random errors when the encoded feature value is decoded from the population by ML estimation. Moreover, total spiking activity in the population is limited by a normalization mechanism (Carandini & Heeger, 2012), meaning that fewer spikes are available to encode each item as set size increases. This allows the model to account quantitatively for the decline in precision with set size: If an item is encoded by fewer spikes, decoding variability and recall error are increased. The error distributions produced by this model show characteristic deviations from the normal distribution with these deviations being particularly evident at larger set sizes. Consistent with this model, we found evidence for non-normally-distributed errors in our location reproduction task.

This neural model has the potential to provide an integrated account for the response latency and precision results in the present task. If fewer spikes are generated to encode the feature of a specific item, this will not only decrease the precision for decoding this feature value, but also lead to a slower accumulation of evidence in response generation. This explains why mean latencies for responses to the target increase with set size (fewer spikes for encoding each item due to normalization), and accounts for the positive correlation between latency and error across individual trials (due to trial-to-trial variability in the number of spikes dedicated to each item). Further modeling work will be necessary to confirm compatibility with this theory, and to link the theory to neural data on the representation of working memory content in the cortex (e.g., Emrich et al., 2013).

Previous work (Jiang, Olson, & Chun, 2000; see also Treisman & Zhang, 2006) has argued that the locations of visual stimuli are stored in relative, rather then absolute, coordinates. This conclusion was based on the observation of poorer performance on change detection tasks when non-target locations were varied between presentation and test. In the present study, using a reproduction method, none of the memory items were presented in the probe display, precluding the use of information about the location of items relative to each other. However, the central fixation may have acted as a stable cue relative to which item locations could be stored.

In this study we used relatively long (2 sec) exposures to the memory array. Previous studies have demonstrated that shorter exposures can disrupt recall performance due to incomplete encoding of the memory array, resulting in increases in both swap errors and guessing (Bays et al., 2009, Bays et al., 2011, Emrich and Ferber, 2012). However, this is unlikely to be the critical difference that explains the discrepancy between our results and those of Zhang and Luck (2008): the previous study tested both short (100 msec) and long (1 sec) presentations and observed a similar plateau in representational precision in both cases.

So why did Zhang & Luck observe a plateau of precision in their color recall task? A key consideration is that the estimate of precision in the mixture model is based on the width of the normal component of the mixture distribution. If the true distribution deviates from the normal distribution, the precision measure obtained from the model may not be a valid estimator of the true precision: in particular, if the distribution is long-tailed then it will systematically underestimate variability. There is now substantial evidence that the true distribution of errors deviates substantially from the normal distribution for simple visual features such as color and orientation, becoming long-tailed at larger set sizes. This non-normality has been explained as a consequence of variability in precision (double-stochasticity; van den Berg et al., 2012, Fougnie et al., 2012), and more mechanistically as a result of the way in which information is represented in neuronal spiking activity (Bays, 2014). Model fits taking into account these sources of non-normality are consistent with a monotonic increase of variability with set size, without a plateau.

The present study found some evidence for non-normality of error distributions at higher set sizes, but overall deviations from normality were comparatively small, resulting in no significant advantage of a mixture model with stable distributions over the normal model. If the normal distribution provides a close approximation for the error distribution in the case of spatial recall, this may explain why we were able to observe a monotonic increase in variability with set size even when using a mixture model with normal distributions (in contrast to the findings in color recall tasks described above). The finding that error distributions are close to normal in this task presents a challenge to variable precision models (Fougnie et al., 2012, van den Berg et al., 2012), which assume that double-stochasticity, and hence non-normality, is a fundamental element of working memory representation. In contrast, it is compatible with the spiking model of Bays (2014), in which the non-normality of the error distribution is a function of both the gain (mean activity) of the neural population encoding the stimuli and the width of neurons' tuning curves. It is possible in this model that internal representation of object locations occurs in a spiking regime, for example one of high gain, in which deviations from normality are small. This is consistent with the observation that non-normality becomes increasingly evident as set size is increased (and hence per-item activity decreased), and it predicts that non-normality will become even more pronounced at set sizes higher than the maximum tested in the present study.

In summary, examining visuospatial recall, we found no evidence for plateaus in working memory precision or retrieval latency that would correspond to reaching a fixed limit on the number of items stored. These results argue against quantization of the representational medium into a small number of “slots”, and instead favor a continuous resource model of working memory.
