# Supplementary material for: No fixed item limit in visuospatial working memory
Source: Cortex. 2016 Oct;83:181–93. doi: 10.1016/j.cortex.2016.07.021 (PMC5043407; doi:10.1016/j.cortex.2016.07.021)
Supplement: Supplementary file 1 [file mmc1.pdf]

Supplementary Material

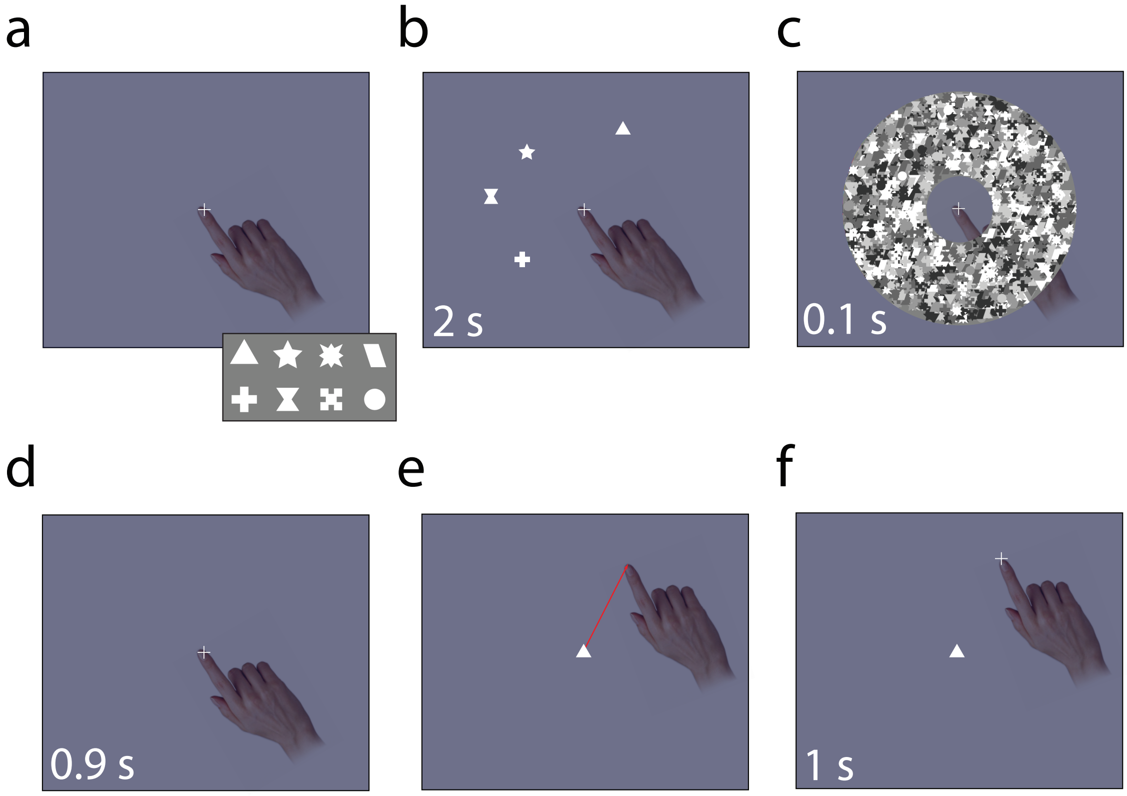

Supplementary Figure 1: Replication task. (a–f) On each trial, a sample array was presented consisting of one to eight distinct shapes at randomized locations, followed by a mask. After a delay, a single shape was presented and the participant moved their fingertip to the corresponding remembered position. The set of shapes used is shown in inset of a.

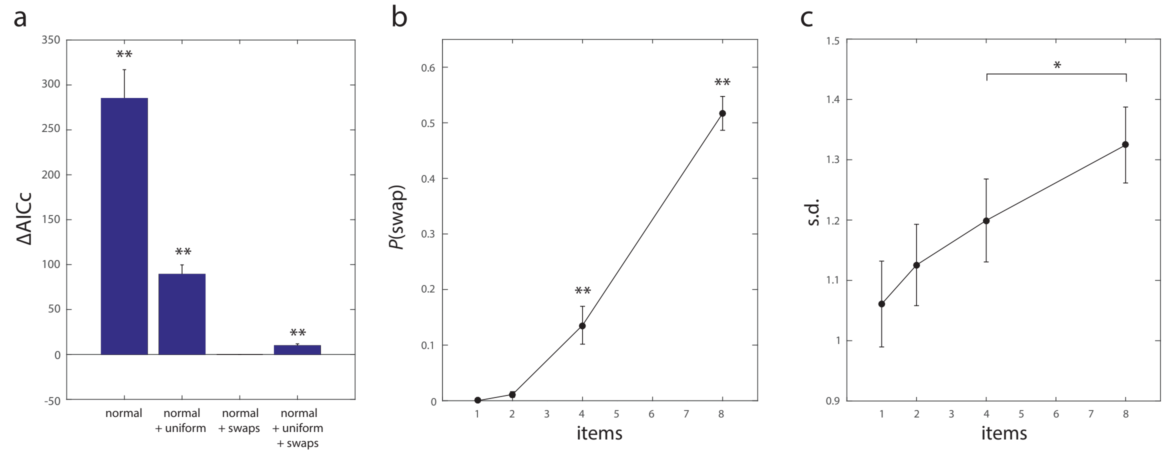

Supplementary Figure 2: Model comparison and parameters of mixture model fit for replication task. (a) AICc scores of different mixture model variants relative to the score of the best fitting model. (b) Estimated proportions of swap errors in best-fitting mixture model for different set sizes. (c) Estimated standard deviations of normal distribution in best-fitting mixture model.

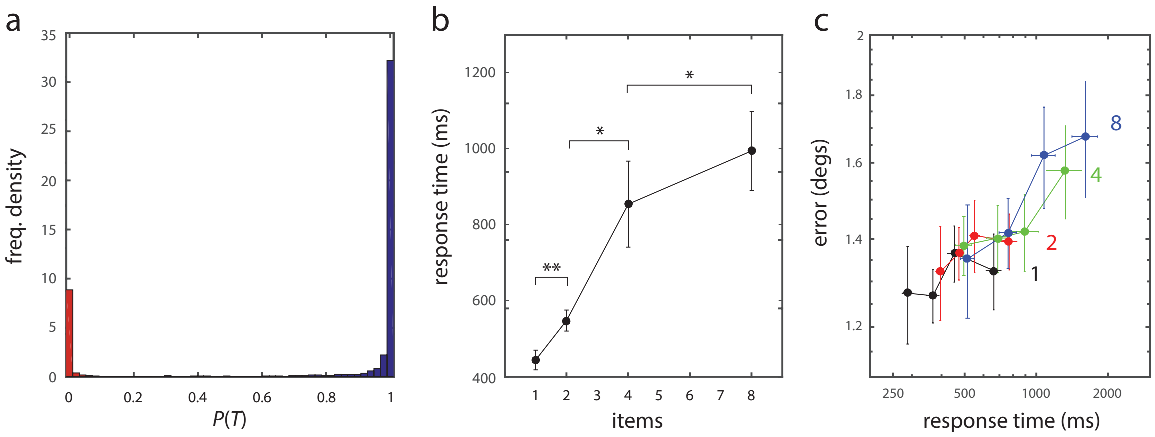

Supplementary Figure 3: Analysis of response latencies for replication task. (a) Distribution of probabilities $P(T)$ that the response of a trial was directed towards the target disc. Trials with $P(T)$ >0.5 are classified as responses to target (blue), all other trials as swap errors (red). (b) Mean response times for different set sizes. Error bars indicate the standard error over subjects. (c) Mean response errors plotted against response times. Trials are quantilized by response time. Filled circles show mean response latency and mean response error for each quantile, error bars indicate standard error in both dimensions.
